# Impacts of 120 years of fertilizer addition on a temperate grassland ecosystem

**DOI:** 10.1371/journal.pone.0174632

**Published:** 2017-03-28

**Authors:** Jonathan Kidd, Peter Manning, Janet Simkin, Simon Peacock, Elizabeth Stockdale

**Affiliations:** 1 School of Agriculture, Food and Rural Development, Newcastle University, Newcastle upon Tyne, United Kingdom; 2 Crop and Soil Systems, Scotland’s Rural College (SRUC), Craibstone Estate, Aberdeen, United Kingdom; 3 Senckenberg Gesellschaft für Naturforschung, Biodiversity and Climate Research Centre (BiK-F), Senckenberganlage 25, Frankfurt, Germany; USDA Agricultural Research Service, UNITED STATES

## Abstract

The widespread application of fertilizers has greatly influenced many processes and properties of agroecosystems, and agricultural fertilization is expected to increase even further in the future. To date, most research on fertilizer impacts has used short-term studies, which may be unrepresentative of long-term responses, thus hindering our capacity to predict long-term impacts. Here, we examined the effects of long-term fertilizer addition on key ecosystem properties in a long-term grassland experiment (Palace Leas Hay Meadow) in which farmyard manure (FYM) and inorganic fertilizer treatments have been applied consistently for 120 years in order to characterize the experimental site more fully and compare ecosystem responses with those observed at other long-term and short-term experiments. FYM inputs increased soil organic carbon (SOC) stocks, hay yield, nutrient availability and acted as a buffer against soil acidification (>pH 5). In contrast, N-containing inorganic fertilizers strongly acidified the soil (<pH 4.5) and increased surface SOC stocks by increasing the C stored in the coarse (2.8 mm-200 μm) and fine (200–50 μm) fractions. Application of N fertilizers also reduced plant species richness and the abundance of forbs and legumes. Overall, our results were broadly consistent with those observed in other very long-term studies (the Park Grass and Steinach Grassland experiments) in that fertilization effects on plant and soil properties appeared to be driven by differences in both nutrient input and changes to soil pH. We also established that the direction of long-term fertilization effects tended to be comparable with short-term experiments, but that their magnitude differed considerably, particularly where ammonium sulphate-induced acidification had occurred. We therefore conclude that short-term studies are unlikely to possess the required timeframe to accurately predict long-term responses, thus necessitating the use of long-term study sites. Such experiments should be strategically established in regions where future fertilizer use is expected to increase rapidly.

## Introduction

Over the last century, agricultural production and a growing human population have become heavily dependent on the use of fertilizers. Key technological developments at the turn of the 20^th^ century supported this, most notably the Haber-Bosch process, resulting in >10-fold increase in the use of reactive nitrogen (N) over the past 150 years [1]. World fertilizer consumption projections suggest this pattern in fertilizer use is unlikely to diminish, with 263 million tonnes of fertilizer expected to be used annually by 2050, an approximate total increase of 60 million tonnes on the present day [2].

The critical contribution that fertilizer application has made in increasing plant productivity, and consequently agricultural yields, has long been recognized [3, 4]. However, the provision of plant-growth limiting nutrients has had wide ranging consequences for many key ecosystem processes and services beyond simply increasing aboveground biomass, including dramatic alterations of plant and soil communities and the processes they control [5–8].

Fertilizer-induced changes to soil properties are numerous and include changes in soil nitrogen cycling [9] and the stocks of soil organic carbon (SOC) found in a range of size and density fractions [10, 11]. Fertilizer additions also often result in a decline in plant species richness [12, 13] and changes in community structure and functional composition [12, 14]. These aboveground changes are accompanied by changes to the microbial community, with effects on soil enzyme activity [15], microbial biomass [16, 17] and microbial composition [18, 19].

Fertilizer-induced shifts to ecosystem functions and services have led to questions about the sustainability of current and future agricultural fertilizer management [20–22], due to problems including; widespread nutrient leaching, groundwater contamination, eutrophication, biodiversity declines and soil acidification. Assessing this potential risk is particularly pertinent in developing countries where fertilizer use per hectare is expected to increase in order to provide food for larger and wealthier populations [23]. Furthermore, despite the fact that in many parts of the developed world, fertilization has been regular practice for many decades, the long-term impacts of this practice have not been fully quantified due to the difficulty in obtaining reliable data on historic rates of addition.

Uncertainty regarding the long-term impacts of fertilizer addition upon ecosystems has partially arisen from a general reliance on relatively short-term experimental studies (typically <10 years) e.g. [15, 24, 25]. While understanding the short-term impacts of fertilization is relevant in situations where management practices are changing i.e. in rotational cropping systems or areas of the developing world where fertilizers are only now starting to be applied, these effects may not be representative of the long-term response of sites remaining under intensive management. As an increase in the number of repeatedly fertilized sites is expected in the future, it may therefore be misleading to extrapolate the effects from short-term studies when predicting long-term ecosystem responses. Ecosystem properties have been shown to take decades to stabilize in response to fertilization [26, 27], meaning that short-term studies can be unrepresentative of long-term responses. While studies that compare differences in ecosystem properties between short- and long-term experiments are scarce, inconsistencies are likely to exist. This may include a failure to detect critical environmental thresholds, beyond which change in ecosystem properties have been shown to be dramatic [28] or gradual species adaptations, such as those evidenced in *Anthoxanthum odoratum* species, both of which were identified at the very long-term Park Grass experiment (160 years) [27]. In addition, transient effects of fertilization on soil C storage may be observed in short-term studies in labile soil C fractions, yet the influence on the more stable C fractions which possess a longer turnover time [29] may only be detected using long-term experiments. Variation between short- and long-term studies may also occur as the magnitude of fertilization effects may differ depending on the length of the experiment. For example, the reduction in the microbial biomass abundance was shown to be greater over longer periods of N fertilization [16], a pattern which short-term experiments would be unlikely to detect. In addition, observational studies can also deliver potentially misleading results due to confounding management effects, e.g. the strong association between increased fertilizer inputs and other components of agricultural intensification [30]. The use of long-term field experiments is consequently of great value. However, the number of long-term ecological studies is very low [27], thus making it hard to draw general conclusions.

In the very few studies that have examined the impacts of long-term fertilization, strong shifts in ecosystem properties are attributed to differences in both the provision of nutrients and soil pH. For instance, in both the Park Grass and the Steinach Grassland (83 years) experiments, plant species richness is not only negatively affected by increased biomass production in response to increased nutrient supply, but also by intense acidification following the addition of ammonium sulphate fertilizer which causes the exclusion of species unable to tolerate highly acidic conditions [27, 31, 32]. In terms of soil properties, concentrations of plant available nutrients are increased by the application of N, P and K containing fertilizers and reduced via uptake by the aboveground biomass [33]. However, nutrient availability is also mediated by differences in soil pH, whereby the nutrient content is reduced in acid soils [34]. Up to now, it has not been established if these long-term trends are general responses of grasslands to fertilization.

To address this, we carried out a comprehensive evaluation of the ecosystem responses of grassland to very long-term fertilizer addition using Palace Leas Hay Meadow; the second oldest continuous grassland experiment in the world [35]. The experiment was established in 1896 to identify means of improving grassland yield and aftermath growth using fertilizers, liming materials and manures. However, numerous other parameters have been measured subsequently [35–38]. Previous research at the site has involved the study of long–term treatment effects on above- and below-ground properties, but studies have tended to report the effects on only a small number of plant and soil properties, and this has been done separately [39, 40] and from an inconsistent set of available plots. The overarching aim of this study was to characterize the responses of a wide range of soil and plant properties i.e. hay yield, plant community composition and soil chemical properties (pH, available P and K and SOC stocks), to long-term fertilizer application in order to (i) establish the Palace Leas experiment as a platform for more specific future research (e.g. into the mechanisms underlying these responses, and as a data-set to contribute to further meta-analyses) and (ii) to compare these responses to those observed at other long-term fertilized sites and with results from short-term studies. We hypothesized, based on previous findings from long-term experiments [27, 31, 32], that (i) changes to plant and soil properties will be driven by direct nutrient input and via changes in soil pH, and that (ii) responses observed at Palace Leas to fertilizer addition will be consistent with those from other long-term grassland experiments, but differ considerably from the findings of short-term studies where plant and soil properties are primarily controlled by differences in the provision of nutrients.

## Materials and methods

### Field site

**Fig 1:**
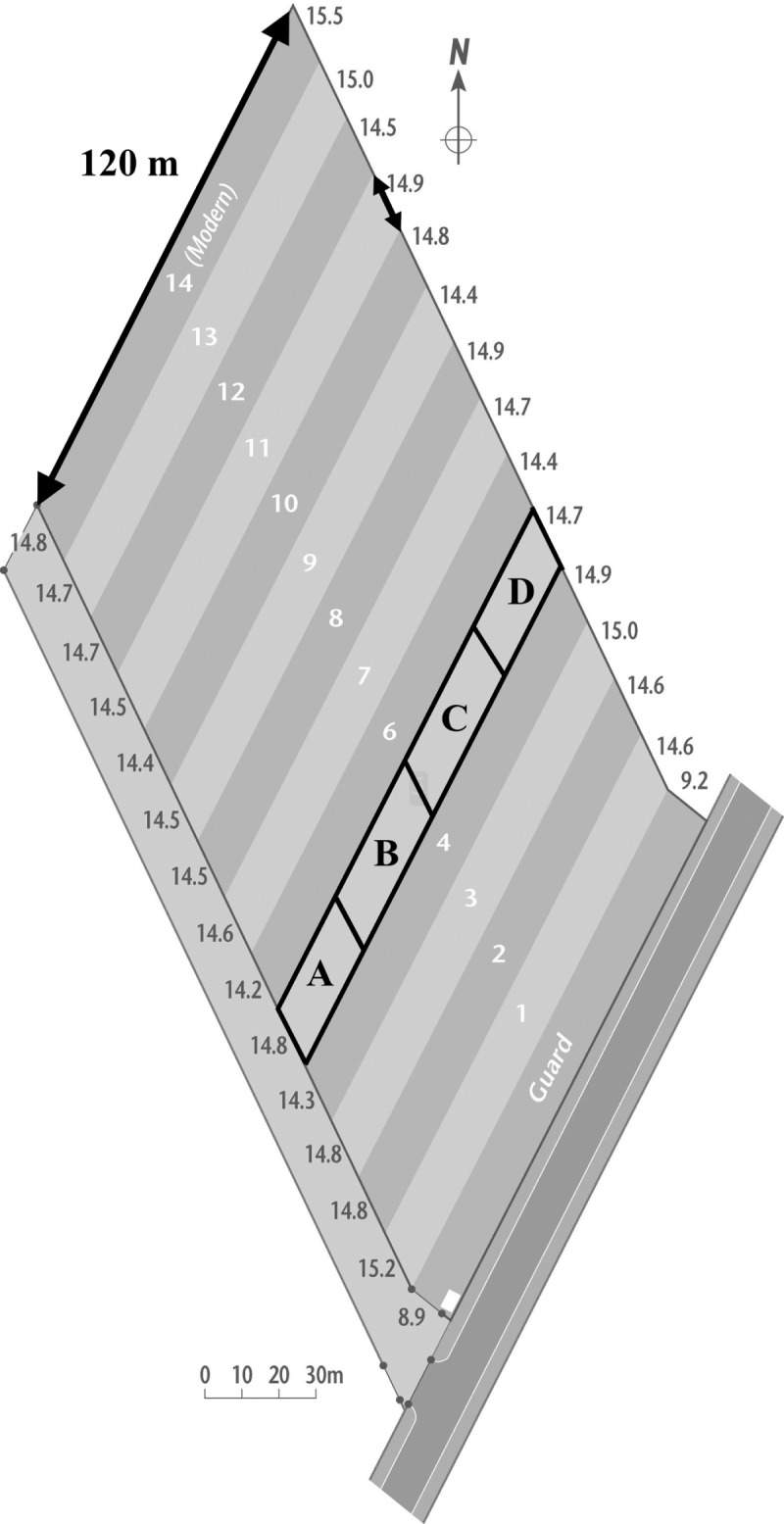
Palace Leas Hay Meadow Experiment layout. Illustration in plot 5 of how the treatment plots were divided into the four blocks; A-D. The length (northern edge) and individual treatment plots widths (eastern and western edge) are labelled in the diagram.

Palace Leas Hay Meadow Experiment is located 30 km north of Newcastle upon Tyne, England at Cockle Park Farm, (55°13' N, 1°41' W, UK National Grid Reference NZ 202912). The soil was classified as a pelo-stagnogley (Typic Ochraqualf) from the Hallsworth series [35] and has a clay loam texture [41] with 41% sand, 29% silt and 30% clay. Prior to the experiment, the vegetation at Palace Leas was permanent pasture for many years [42]. Due to its close proximity to the farm, the field had regularly received farmyard manure (FYM). Using data from a botanical survey carried out in 1897 (year 2 of the experiment), the initial grassland vegetation on all treatment plots was matched to the National Vegetation Classification [43] community U4b (*Festuca ovina-Agrostis capillaris-Galium saxatile* grassland, *Holcus lanatus-Trifolium repens* sub-community) [44]. As it can take many years for grassland communities to respond to change in management, it is reasonable to assume that Palace Leas would have been U4b before the experiment was set up. The experiment was established in 1896 in a *c*. 2 ha grassland field and was arranged as 14 parallelogram strips each *c*. 120 x 15 m (Fig 1). There was also a guard strip which stretched along the southern edge of the site, parallel to the road. The establishment of the experiment predated the use of replicated experimental designs for several decades [45], and so at Palace Leas the fertilizer treatments were not replicated or randomized. The 14 fertilizer treatment plots consisted of five applied with varying amounts of cattle FYM, some of which also received N, phosphorus (P) and potassium (K), eight plots that received mineral fertilizer treatments comprising of all combinations of N, P and K fertilizers and an unfertilized control (Table 1). The FYM was applied in February while mineral fertilizers were applied in late March or early April. In terms of total fertilizer addition, plots treated with FYM generally received higher rates of nutrient application than those treated with mineral fertilizer (Table 1). The experiment has remained under constant management since 1896 with the exception of plot 14 (_H_NPK), which was added in 1976 and received a higher rate of N, P and K (Table 1), typical of modern fertilizer management. Among the treatment plots that were applied with mineral fertilizer only, those that received N did so in the form of ammonium sulphate ((NH_4_)_2_SO_4_), with the exception of _H_NPK which received ammonium nitrate (NH_4_NO_3_). Where both FYM and mineral fertilizer was applied, mineral fertilizer N was a 50:50 split of ammonium sulphate and sodium nitrate (NaNO_3_). The form of K applied throughout the experiment was muriate of potash (KCl). The only change to the existing treatments throughout the history of the experiment was the form of P applied, which was modified in 1976 from basic slag [(CaO)_5_ P_2_O_5_ SiO_2_] to triple superphosphate (Ca(H_2_PO_4_)_2_^.^H_2_O) as the phosphate content in slag was becoming too variable. An annual hay cut was taken in July since the start of the experiment in order to determine hay yield in each treatment plot. Cattle or sheep were allowed to graze freely the post-cut aftermath growth in late summer and again briefly in winter which allowed for the potential transfer of nutrients via dung across the site. Permission for sampling was granted by the School of Agriculture, Food and Rural Development at Newcastle University. No endangered or protected species were involved in the study.

**Table 1:** Details of the fertilizer treatments at Palace Leas Hay Meadow Experiment.

Plot number	Treatment	Details	Total nutrient addition (kg ha^-1^ y^-1^)			
			C	N	P	K
**1**	FYM + NPK	20 t ha^-1^ farm-yard manure, 17 kg N ha^-1^, 30 kg P ha^-1^ and 34 kg K ha^-1^ of inorganic fertilizer applied annually	800	137	94	194
**2**	FYM	20 t ha^-1^ farm-yard manure applied annually	800	120	64	160
**3**	FYM + NPK	20 t ha^-1^ farm-yard manure applied in year 1 and 17 kg N ha^-1^, 30 kg P ha^-1^ and 34 kg K ha^-1^ of inorganic fertilizer applied in year 2	400	69	47	97
**4**	FYM	20 t ha^-1^ farm-yard manure applied every other year	400	60	32	80
**5**	FYM + NPK	40 t ha^-1^ farm-yard manure applied in year 1 and 17 kg N ha^-1^, 30 kg P ha^-1^ and 34 kg K ha^-1^ of inorganic fertilizer applied in years 2, 3 and 4	400	73	55	106
**6**	Control	No fertilizer applied	0	0	0	0
**7**	N	35 kg N ha^-1^ of inorganic fertilizer applied annually	0	35	0	0
**8**	P	60 kg P ha^-1^ of inorganic fertilizer applied annually	0	0	60	0
**9**	K	67 kg K ha^-1^ of inorganic fertilizer applied annually	0	0	0	67
**10**	NP	35 kg N ha^-1^ and 60 kg P ha^-1^ of inorganic fertilizer applied annually	0	35	60	0
**11**	NK	35 kg N ha^-1^and 67 kg K ha^-1^ of inorganic fertilizer applied annually	0	35	0	67
**12**	PK	60 kg P ha^-1^ and 67 kg K ha^-1^ of inorganic fertilizer applied annually	0	0	60	67
**13**	NPK	35 kg N ha^-1^, 60 kg P ha^-1^ and 67 kg K ha^-1^ of inorganic fertilizer applied annually	0	35	60	67
**14**	NPK	100 kg N ha^-1^, 66 kg P ha^-1^ and 100 kg K ha^-1^ of inorganic fertilizer applied annually	0	100	66	100

Total carbon and nutrient addition was calculated as the average over a 4 year period and included nutrient content in the FYM and inorganic fertilizer. Total nutrient content in the cattle FYM was estimated using the DEFRA Fertiliser Manual (RB209) [46], typical totals for each nutrient = 6 kg N t^-1,^ 3.2 kg P t^-1^ and 8 kg K t^-1^ (fresh weight). The approximate C content of FYM added to plots was estimated assuming FYM is *c*.40% C when dry [35].

### Soil sampling

Soil samples were collected in September 2013 from all 14 plots at four replicate positions across each plot; 15 m (A), 45 m (B), 75 m (C) and 105 m (D) (Fig 1). Five cores (5 cm depth, 5 cm diam.) were collected within 30 cm of each replicate sampling point at 0–5 cm and 5–10 cm depths using a manual borer. Soil cores taken from the same depth and sampling point were bulked and homogenized. In plots 7 and 11 the presence of a 6 cm deep organic surface layer [47], which was absent in other plots, meant that cores were also taken from this O horizon and at 0–5 cm and 5–10 cm from the mineral soil below.

### Soil properties

Soil pH was determined using a 1:2.5 soil-water suspension. Soil water content and bulk density were determined by oven drying 10 g (2.8 mm sieved) of moist soil at 105°C. Total soil C and N concentrations were determined on a 0.1 g (dry weight equivalent; DW) sample via dry combustion using a Vario Macro Cube (Elementar, Hanau, Germany) and converted to kg C and N per m^2^ by multiplying the C and N concentrations by the bulk density and the thickness of the soil layer.

A modified wet sieving method was used to measure C concentration in the soil particle-size fractions [48]. In brief, 10 g (DW) of each soil sample was mixed with 50 ml of deionized water, agitated for 16 h with glass beads and the solution was poured through a series of sieves (2.8 mm, 200 μm and 50 μm meshes). Each of the soil samples were sieved by hand for 30 min, the remaining solutions (<50 μm) were vacuum filtered through 0.45 μm membrane filters to isolate the very fine fraction from dissolved organic C. All fractions were dried at 40°C until constant weight was obtained. The 2.8 mm-200 μm (coarse carbon fraction) and 200–50 μm (fine carbon fraction) fractions were weighed while the weight of the 50–0.45 μm (very fine carbon fraction) was obtained from the sum of the coarse carbon and fine carbon fractions subtracted from the initial soil mass (10 g). Soil fractions were then ground in a ball mill. The C concentration of each fraction was determined on a 0.1 g dried sample via dry combustion using a Vario Macro Cube and transformed into stocks (kg C per m^2^) using the same calculation as used for total C and N stocks.

Available P was extracted using the Olsen-P method [49] and analyzed using molybdate-blue colour determination [50]. Exchangeable K was extracted using 1 mol L^-1^ ammonium nitrate solution and determined using flame photometry [51]. Exchangeable Al was determined via a 1 mol L^-1^ KCl extraction, a titration using 0.01 mol L^-1^ NaOH and a back titration using 0.01 mol L^-1^ HCl [52].

### Plant analysis

The annual hay cut was taken in early July, a 10 m^2^ sample was obtained from the middle of each block (A-D) of each treatment plot, at the same position as where the soil samples were collected. From this a fresh sub sample was taken, dried at 80°C for 24 h and reweighed to determine dry matter yield. Hay yield data (recorded from 1896–2015) is stored in the Palace Leas archive, a data repository containing all recorded data from the experiment. Long-term mean hay yield for each of the 14 fertilizer treatment plots was determined using archived data from 1976–2015. For the years 1982, 1985 and 1997, replicate block hay yield values were not recorded, only plot level mean yields were available (S1 Dataset). An 80 g (DW) sample from the 2015 hay cut from each replicate sampling point was milled to a fine homogenized powder and a 0.1 g subsample was analyzed for C and N concentrations using the aforementioned Vario Macro Cube.

On 5^th^ June 2015 a full botanical survey was undertaken at the site. In each treatment plot, quadrats were laid out at four points which approximately corresponded to the same location at which soil samples were obtained (A, B, C and D; Fig 1). An additional quadrat was taken in the middle of the plot (between B and C) so that five quadrats were available to allow reliable classification of the vegetation according to the UK National Vegetation Classification (NVC). Percentage cover for all vascular plants and bryophytes was recorded for a 1 m^2^ quadrat at each point. Species present at <1% cover were recorded as 0.1%.

### Data analysis

Measured soil and plant parameters from the four sampling points of each plot were kept separate to give four replicates per plot. One-way ANOVA was then used to compare differences in the measured soil and plant parameters between treatment plots, rather than between replicated treatments, as would be typical in modern replicated and randomized field trials. Significant differences between treatment plots were tested using Tukey’s post-hoc comparisons of means at significance level *P*<0.05. Before ANOVA was performed, data was checked for normality and equal variance, data was log-transformed where necessary. Where the criteria for ANOVA could not be met by transformation (Olsen-P, exchangeable K, exchangeable Al, SOC at 5–10 cm, legume cover and bryophyte cover), Kruskal-Wallis tests followed by Dunn’s test for multiple comparisons with Bonferroni corrections were performed. The O horizon results from plots 7 and 11 were omitted from ANOVA (see S1 Table). Pearson correlation coefficient was used to explore relationships between soil and vegetation properties. ANOVA, Kruskal-Wallis tests and Pearson correlation coefficients were computed using R version 3.2.1 [53].

Principal components analysis (PCA) was performed using Canoco version 4.5 and Canodraw [54] to identify the major patterns among the plant species. Treatment plot numbers and environmental data, including selected soil and plant parameters, were overlain on the ordination biplot to help explain trends in plant species abundance. Plant species richness was measured as the total number of vascular and non-vascular plant species present in each quadrat. Plant and bryophyte species were grouped into four functional groups; grasses (including sedges and rushes), legumes, forbs and bryophytes. One-way ANOVA was used to assess fertilizer treatment plot effects on the cover of the functional groups and plant species richness.

For the NVC, five quadrats from each treatment plot of the botanical survey were combined for analysis. Doing this accounted for the patchiness of the vegetation due to ridge and furrow originating from historic cultivation at the site. The fit of each plot to defined sub-communities in the NVC was then calculated using TableFit version 2 [55], and published descriptions [43].

## Results

### Soil properties

#### Soil pH

**Fig 2:**
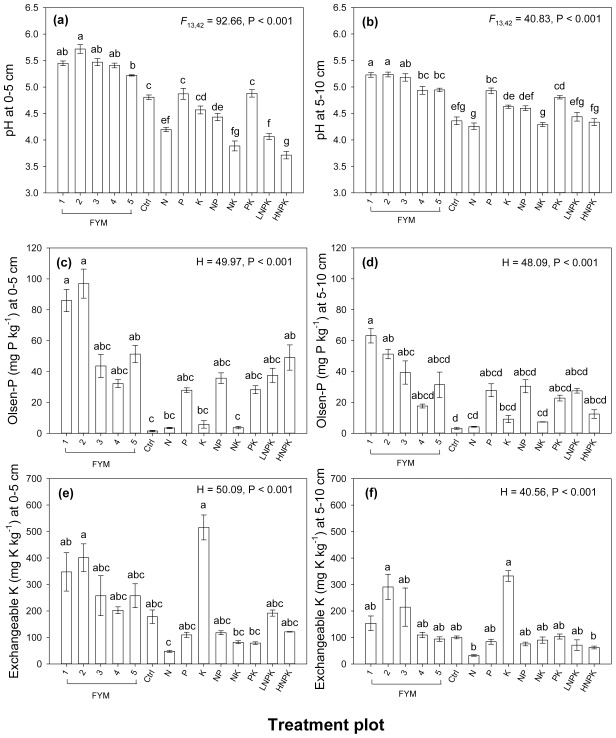
**Effect of fertilizer treatment plot on (a) soil pH at 0–5 cm, (b) soil pH at 5–10 cm, (c) Olsen-P at 0–5 cm, (d) Olsen-P at 5–10 cm, (e) exchangeable K at 0–5 cm and (f) exchangeable K at 5–10 cm.** Error bars represent ±1 standard error of the treatment plot mean and contrasting letters denote significant differences between treatment plots. H is the Kruskal-Wallis test statistic.

Soil pH ranged from 3.17 to 5.72 in the soil at 0–5 cm, 5–10 cm and in the O horizon (Fig 2A and 2B, S1 Table). Soil pH differed significantly between fertilizer treatment plots; plots applied with FYM (10 and 20 t ha^-1^) had significantly higher pH (range 4.94–5.72) than those receiving mineral fertilizer (range 3.17–4.93), with the exception of the plots treated with P (plot 8) and PK (plot 12) at 5–10 cm (Fig 2A and 2B, S1 Table). Soil pH decreased with depth in plots where FYM was applied (plot 1–5) and in the control (plot 6) but increased with depth in plots applied with mineral fertilizer only (plot 7–14), with the exception of PK (Fig 2A and 2B), thus indicating acidifying effects of mineral fertilizers and neutralising effects of FYM on topsoil.

#### Olsen-phosphorus

Olsen-P was higher in plots applied with FYM and soils applied with mineral P fertilizer. In plots where FYM was applied at a higher rate (plot 1 and 2; 20 t FYM ha^-1^), Olsen-P was significantly higher than in the N only (plot 7), K only (plot 9), NK (plot 11) treated plots or the control at both 0–5 cm and 5–10 cm (Fig 2C and 2D). At 0–5 cm in the plot treated with the high rate of NPK (_H_NPK, plot 14) Olsen-P was comparable (49.09 mg P kg^-1^) to that of soils in plots receiving FYM (range 32.22–96.95 mg P kg^-1^), however, it decreased 4-fold at 5–10 cm and was not significantly different from plots not applied with P. Such decreases in Olsen-P with depth were less pronounced in plots receiving FYM (Fig 2C and 2D).

#### Exchangeable potassium

The treatment plots receiving FYM and K only had the highest exchangeable K and were significantly higher than the N only treated plot at 0–5 cm (Fig 2E). Conversely at 5–10 cm, the plots that received the higher rate of FYM only (plot 2) and K only had significantly higher exchangeable K than the plot that received N only (Fig 2F). Similar to Olsen-P, exchangeable K decreased considerably in the _H_NPK treatment plot at 5–10 cm (62.31 kg K ha^-1^), only the N treated plot (32.26 kg K ha^-1^) had lower exchangeable K (Fig 2F).

#### Exchangeable aluminium

Exchangeable Al was higher in the mineral fertilized plots. Soils receiving N only and NK had significantly higher exchangeable Al (range 31.60–47.83 mmol kg^-1^) than soils receiving FYM (range 0–1.12 mmol kg^-1^) at both 0–5 and 5–10 cm, with the exception of the plot applied with FYM every other year (plot 4) at 5–10 cm. In the plot receiving the higher rate of FYM only, exchangeable Al was absent at both 0–5 and 5–10 cm (Tables 2 and 3).

**Table 2:** Total nitrogen content, soil carbon to nitrogen ratio (C/N), coarse fraction carbon stocks, fine fraction carbon stocks, very fine fraction carbon stocks and exchangeable Al for the Palace Leas plots at 0–5 cm depth.

Plot number	Treatment^a^	Total nitrogen content (kg N m^-2^)^b^	Soil C/N^b^	Coarse fraction carbon stocks (kg C m^-2^)^b^	Fine fraction carbon stocks (kg C m^-2^)^b^	Very fine fraction carbon stocks (kg C m^-2^)^b^	Exchangeable Al (mmol kg^-1^)^c^
1	FYM+ NPK	0.28 (0.01) a	11.44 (0.20) ef	0.17 (0.04) b	1.66 (0.25) a	1.21 (0.07) abcdef	0.00 (0.00) c
2	FYM	0.28 (0.01) a	11.00 (0.08) f	0.22 (0.02) ab	1.58 (0.26) a	1.55 (0.12) ab	0.00 (0.00) c
3	FYM + NPK	0.23 (0.02) ab	12.32 (0.30) def	0.20 (0.04) b	1.08 (0.22) ab	1.34 (0.09) abcde	0.75 (0.43) bc
4	FYM	0.22 (0.01) abc	12.05 (0.34) def	0.22 (0.05) ab	1.09 (0.10) ab	1.37 (0.11) abcd	0.74 (0.43) bc
5	FYM + NPK	0.23 (0.01) abc	12.10 (0.49) def	0.20 (0.04) b	1.61 (0.22) a	1.30 (0.05) abcdef	0.12 (0.12) bc
6	Control	0.17 (0.01) bcde	13.49 (0.27) bcde	0.15 (0.02) b	1.25 (0.24) ab	0.92 (0.09) def	4.12 (0.31) abc
7	N	0.15 (0.01) de	15.38 (0.81) ab	0.18 (0.08) b	0.75 (0.06) b	1.62 (0.14) a	47.83 (1.07) a
8	P	0.18 (0.01) bcde	12.74 (0.42) cdef	0.24 (0.10) ab	1.16 (0.11) ab	1.07 (0.08) cdef	4.62 (0.24) abc
9	K	0.17 (0.01) bcde	14.62 (0.39) abc	0.35 (0.07) ab	1.31 (0.14) ab	0.85 (0.08) f	12.70 (1.85) abc
10	NP	0.17 (0.01) bcde	13.38 (0.15) bcde	0.30 (0.13) ab	1.09 (0.12) ab	1.00 (0.03) cdef	18.68 (0.48) abc
11	NK	0.13 (0.02) e	16.23 (0.55) a	0.16 (0.04) b	0.78 (0.06) b	1.44 (0.15) abc	42.03 (4.42) a
12	PK	0.17 (0.02) cde	13.54 (0.51) bcde	0.26 (0.13) ab	0.96 (0.22) ab	1.13 (0.04) bcdef	3.99 (0.79) abc
13	NPK	0.16 (0.01) de	13.96 (0.60) bcd	0.28 (0.06) ab	1.06 (0.06) ab	0.90 (0.04) ef	19.84 (1.79) abc
14	NPK	0.21 (0.01) bcd	13.91 (0.52) bcd	0.68 (0.11) a	1.50 (0.23) a	0.88 (0.09) ef	31.11 (2.01) ab
*F*_13,42_		12.96	11.13	2.23	2.67	7.88	n/a
H		n/a	n/a	n/a	n/a	n/a	52.99
Significance		***	***	*	**	***	***

^a^ see Table 1 for fertilizer treatment details.

^b^ denotes effect significance determined by ANOVA and Tukey’s post-hoc comparisons of means.

^c^ denotes effect significance determined by Kruskal-Wallis tests and Dunn’s test for multiple comparisons. H is the Kruskal-Wallis test statistic. Significance at *P*<0.001;***, *P*<0.01;**, *P*<0.05;*. Contrasting letters denote significant differences between treatment plots. n/a indicates not applicable.

**Table 3:** Total nitrogen content, soil carbon to nitrogen ratio (C/N), coarse fraction carbon stocks, fine fraction carbon stocks, very fine fraction carbon stocks and exchangeable Al for the Palace Leas plots at 5–10 cm depth.

Plot number	Treatment^a^	Total nitrogen content (kg N m^-2^)^b^	Soil C/N^b^	Coarse fraction carbon stocks (kg C m^-2^)^b^	Fine fraction carbon stocks (kg C m^-2^)^b^	Very fine fraction carbon stocks (kg C m^-2^)^b^	Exchangeable Al (mmol kg^-1^)^c^
1	FYM+ NPK	0.23 (0.02) a	11.53 (0.80)	0.08 (0.02)	0.74 (0.11) a	1.54 (0.24) ab	0.25 (0.25) c
2	FYM	0.21 (0.01) ab	13.03 (1.66)	0.07 (0.01)	0.76 (0.11) a	1.62 (0.15) a	0.00 (0.00) c
3	FYM + NPK	0.19 (0.01) abc	12.91 (0.93)	0.07 (0.01)	0.41 (0.05) ab	1.42 (0.06) ab	1.12 (1.12) c
4	FYM	0.21 (0.01) ab	13.86 (0.97)	0.06 (0.01)	0.53 (0.04) ab	1.51 (0.08) ab	0.87 (0.51) bc
5	FYM + NPK	0.21 (0.01) ab	11.73 (0.27)	0.08 (0.02)	0.53 (0.02) ab	1.51 (0.08) ab	0.50 (0.29) c
6	Control	0.17 (0.01) bc	13.26 (0.38)	0.09 (0.02)	0.44 (0.11) ab	1.34 (0.09) ab	12.71 (0.93) abc
7	N	0.11 (0.01) d	15.04 (0.51)	0.11 (0.04)	0.22 (0.02) b	1.08 (0.08) b	33.97 (3.30) a
8	P	0.16 (0.01) bc	12.80 (1.11)	0.06 (0.02)	0.48 (0.05) ab	1.41 (0.08) ab	3.11 (0.71) abc
9	K	0.16 (0.01) bc	14.87 (0.52)	0.06 (0.01)	0.60 (0.15) ab	1.41 (0.16) ab	16.79 (2.81) abc
10	NP	0.16 (0.01) bc	15.23 (1.76)	0.06 (0.01)	0.44 (0.07) ab	1.48 (0.05) ab	11.90 (1.25) abc
11	NK	0.11 (0.01) d	14.97 (0.28)	0.14 (0.04)	0.20 (0.01) b	1.13 (0.07) b	31.60 (1.44) ab
12	PK	0.16 (0.01) bc	15.73 (1.53)	0.05 (0.01)	0.47 (0.10) ab	1.23 (0.03) ab	5.72 (1.16) abc
13	NPK	0.15 (0.01) cd	14.61 (1.00)	0.07 (0.01)	0.50 (0.11) ab	1.11 (0.17) b	14.66 (1.89) abc
14	NPK	0.14 (0.01) cd	15.29 (1.84)	0.07 (0.01)	0.49 (0.08) ab	1.30 (0.03) ab	31.67 (3.99) ab
*F*_13,42_		13.89	1.77	1.29	3.40	2.30	n/a
H		n/a	n/a	n/a	n/a	n/a	51.44
Significance		***	0.08	0.26	**	*	***

^a^ see Table 1 for fertilizer treatment details.

^b^ denotes effect significance determined by ANOVA and Tukey’s post-hoc comparisons of means.

^c^ denotes effect significance determined by Kruskal-Wallis tests and Dunn’s test for multiple comparisons. H is the Kruskal-Wallis test statistic. Significance at *P*<0.001;***, *P*<0.01;**, *P*<0.05;*. Contrasting letters denote significant differences between treatment plots. n/a indicates not applicable.

#### Soil organic carbon stocks

**Fig 3:**
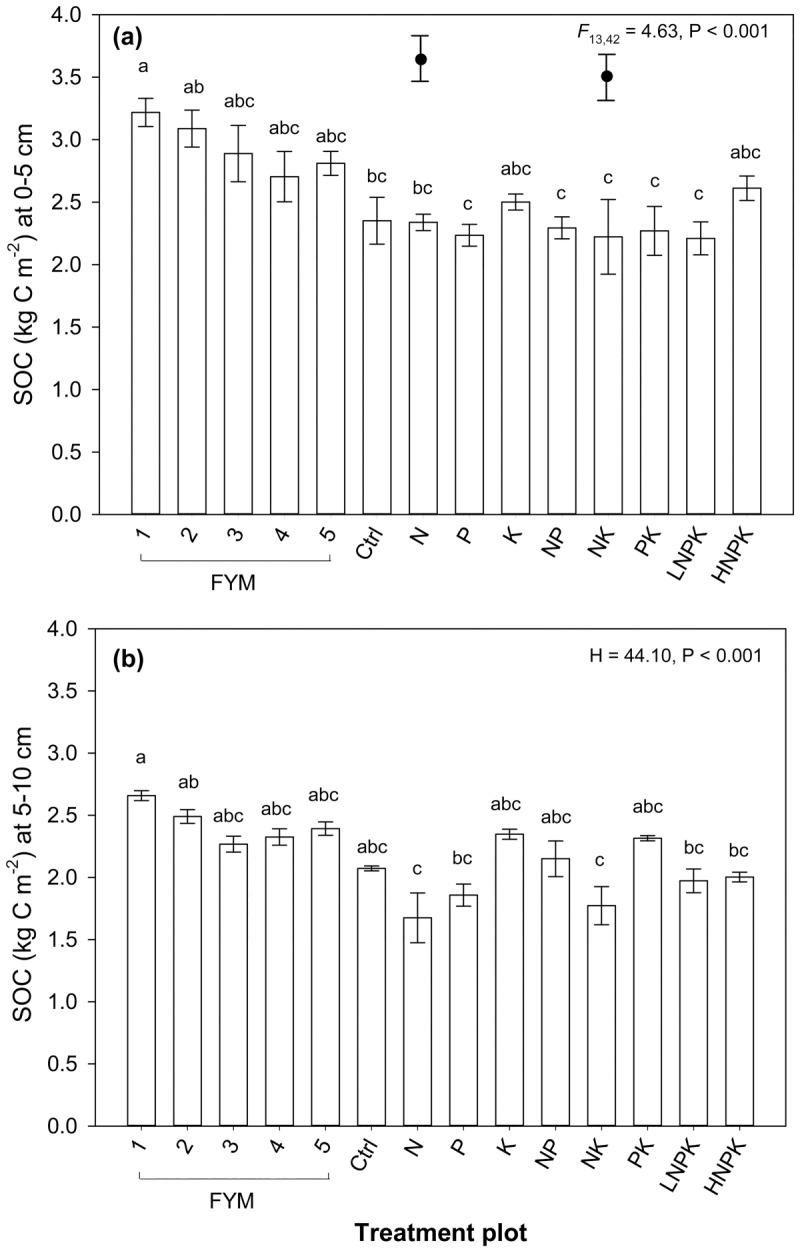
**Effect of fertilizer treatment plot on (a) soil organic carbon stocks at 0–5 cm and (b) soil organic carbon stocks at 5–10 cm.** Error bars represent ±1 standard error of the treatment plot mean and contrasting letters denote significant differences between treatment plots. H is the Kruskal-Wallis test statistic. Filled ellipses with error bars represent the mean and ±1 standard error for the O horizon in plots 7 (N) and 11 (NK).

SOC stocks were highest at low pH in the O horizon of the N and NK treated plots (3.69 and 3.59 kg C m^-2^, respectively; Fig 3A). At 0–5 and 5–10 cm, SOC stocks were higher in plots applied with FYM (range 2.70–3.22 kg C m^-2^ and 2.27–2.66 kg C m^-2^, respectively) than plots where only mineral fertilizer was applied (range 2.21–2.61 kg C m^-2^ and 1.68–2.35 kg C m^-2^ respectively; Fig 3A and 3B). SOC stocks were significantly higher in the plot treated with FYM at a higher rate with NPK than plots receiving N only, P only, NK and the lower rate of NPK (_L_NPK, plot 13) at both 0–5 and 5–10 cm (Fig 3A and 3B). There was a decrease in SOC stocks with depth in all plots except in the PK (+2%) treated plot. The largest reductions in SOC stocks between 0–5 cm and 5–10 cm were in the N, NK and _H_NPK plots (mean = -24%; (Fig 3A and 3B).

In total, 97% (±1.51) of SOC was recovered in particle-size fractionation from 0–5 cm, 5–10 cm and the O horizon. The very fine carbon fraction (50–0.45 μm) contained 52% of the total C stock with 39% and 9% in the fine carbon fraction (200–50 μm) and coarse carbon fractions (2 mm-200 μm), respectively. The _H_NPK plot had significantly higher coarse C fraction stocks at 0–5 cm (0.68 kg C m^-2^) than the N only and NK treated plots and plots receiving both FYM and NPK (plot 1, 3 and 5; Table 2). Similarly, in the other two very acidic N only and NK treatment plots, coarse fraction C stocks were high in the surface O horizon (1.17 and 0.50 kg C m^-2^, respectively; S1 Table). Plots treated with the higher rate of FYM had the highest fine carbon fraction stocks (range 0.74–1.66 kg C m^-2^), conversely the acidic N only and NK treated soils had significantly lower fine fraction stocks (range 0.20–0.78 kg C m^-2^) than plots receiving higher rates of FYM at both 0–5 and 5–10 cm (Tables 2 and 3). Very fine fraction C stocks were higher in FYM amended plots (range 1.21–1.55 kg C m^-2^) and the acidic N only and NK treated soils (range 1.44–1.62 kg C m^-2^) at 0–5 cm (Table 2), however, dissimilar to all other treatment plots very fine fraction C stocks decreased between 0–5 cm and 5–10 cm in the N only and NK plots and were significantly lower than soils receiving the higher rate of FYM only at 5–10 cm (Table 3).

#### Total nitrogen stocks and soil C/N

Total nitrogen stocks differed strongly between treatment plots (Tables 2 and 3) and followed a very similar pattern to SOC as they were highly positively correlated (*r* = 0.91, *P*<0.001 at 0–5 cm, *r* = 0.76, *P*<0.001 at 5–10 cm). Soils with higher rates of FYM application contained significantly more total N (0.28 kg N m^-2^) than soils with mineral fertilizer applied and the control at 0–5 cm (range 0.13–0.23 kg N m^-2^; Table 2), whilst at 5–10 cm only the plot treated with FYM at a higher rate with NPK contained significantly more total N (0.23 kg N m^-2^) than soils applied with mineral fertilizer and the control (range 0.11–0.21 kg N m^-2^; Table 3). At 0–5 and 5–10 cm, the N only (0.15 and 0.11 kg N m^-2^, respectively) and NK (0.13 and 0.11 kg N m^-2^, respectively) treated plots had significantly lower total N than all plots treated with FYM (0–5 cm range 0.22–0.28 kg N m^-2^, 5–10 cm range 0.19–0.23 kg N m^-2^; Tables 2 and 3). In _H_NPK, 70% of the total N was found in the top 5 cm (0.21 kg N m^-2^); similar to the pattern observed in the N only and NK, in which N was considerably higher in the O horizon (0.24 and 0.31 kg N m^-2^, respectively; S1 Table). The acidic N only and NK treated plots also had significantly higher soil C/N (15.38 and 16.23, respectively) than those plots receiving FYM (range 11.00–12.32) at 0–5 cm but soil C/N did not differ significantly between plots at 5–10 cm (range 11.53–15.73; Tables 2 and 3).

### Plant properties

#### Hay yield and hay C/N

**Fig 4:**
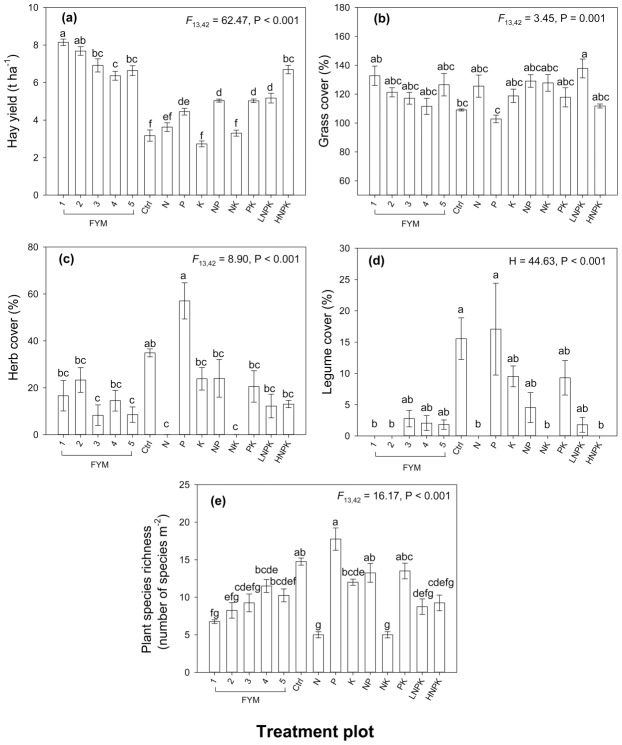
**Effect of fertilizer treatment plot on (a) hay yield, (b) grass cover, (c) herb cover, (d) legume cover and (e) plant species richness.** Error bars represent ±1 standard error of the treatment plot mean and contrasting letters denote significant differences between treatment plots. H is the Kruskal-Wallis test statistic.

**Fig 5:**
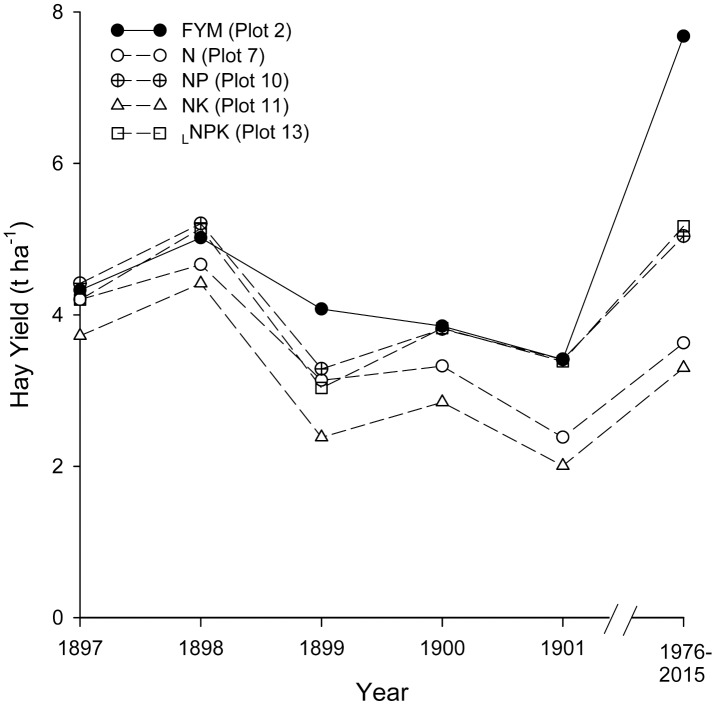
Changes in mean hay yield at Palace Leas for the FYM (20 t ha^-1^) and four ammonium sulphate-containing fertilizer treatments for the first 5 years of the experiment (1897–1901) and the more recent long-term average (1976–2015).

Mean hay yield across years (1976–2015) and hay C/N ratio differed significantly between fertilizer treatment plots (Fig 4A; Table 4). Hay yield was highest in plots applied with FYM and with the exception of _H_NPK (6.69 t ha^-1^) was significantly higher (range 6.37–8.15 t ha^-1^) than where only mineral fertilizer was applied (range 2.73–6.69 t ha^-1^) or in the control plot (3.17 t ha^-1^). Hay yield was significantly higher in the plot treated with FYM at a higher rate with NPK (plot 1) than all other plots with the exception of the plot that received the high rate of FYM only (plot 2). The K only treated plot had the lowest hay yield, producing less than half of the biomass grown by plots receiving FYM or the _H_NPK (Fig 4A). Despite notable long-term differences in hay yield between plots applied with FYM and those receiving only mineral fertilizer, at the beginning of the experiment there was only relatively small differences between the treatment plots (Fig 5). Hay C/N was significantly lower in the _H_NPK than in the NP (plot 10), P only, control, FYM every other year and PK treatment plots (Table 4).

**Table 4:** Hay C/N ratio, bryophyte cover and National Vegetation Classification (NVC) in each fertilizer treatment plot.

Plot number	Treatment^a^	Hay C/N^b^	Bryophyte cover (%)^c^	NVC
1	FYM + NPK	34.71 (2.09) ab	0.00 (0.00)	MG7d
2	FYM	35.24 (1.62) ab	0.00 (0.00)	MG7d
3	FYM + NPK	34.60 (1.18) ab	0.00 (0.00)	MG7d
4	FYM	36.06 (2.13) a	0.00 (0.00)	MG7d
5	FYM + NPK	34.51 (1.58) ab	0.00 (0.00)	MG7d
6	Control	36.64 (2.14) a	0.03 (0.02)	MG5a
7	N	33.39 (1.47) ab	0.00 (0.00)	U4b
8	P	36.96 (1.50) a	0.00 (0.00)	MG5a
9	K	35.65 (1.14) a	0.25 (0.25)	U4b
10	NP	36.96 (0.64) a	0.03 (0.03)	U4b
11	NK	33.20 (2.22) ab	0.00 (0.00)	U4b
12	PK	35.78 (2.01) a	0.00 (0.00)	U4b
13	NPK	35.15 (2.56) ab	0.00 (0.00)	U4b
14	NPK	26.68 (0.53) b	0.00 (0.00)	U4b
*F*_13,42_		2.23	n/a	n/a
H		n/a	11.42	n/a
Significance		*	0.58	n/a

^a^ see Table 1 for fertilizer treatment details.

^b^ denotes effect significance determined by ANOVA and Tukey’s post-hoc comparisons of means.

^c^ denotes effect significance determined by Kruskal-Wallis tests and Dunn’s test for multiple comparisons. Values are means ± 1 s.e. H is the Kruskal-Wallis test statistic. Significance at *P*<0.001;***, *P*<0.01;**, *P*<0.05;*, *P*>0.05; NS. n/a indicates not applicable. Contrasting letters denote significant differences between treatment plots.

#### National vegetation classification

Fertilizer treatment plots were matched to one of two broad NVC categories; mesotrophic grasslands (MG) or calcifugous grassland communities (U). Plots where FYM was applied, the control and P only treatment plots were classified as mesotrophic grasslands, but their closest fit was to two different NVC communities. Those plots receiving FYM were closest to MG7d (*Lolium perenne*—*Alopecurus pratensis* grassland), which is typical of hay meadows on fertile, moist soils. In contrast, the control and the P only treatment plots were closest to MG5a (*Cynosurus cristatus - Centaurea nigra* grassland, *Lathyrus pratensis* sub-community), which is typical of traditionally managed grazed hay meadows (Table 4). Where plots were applied with mineral fertilizers, with the exception of the P only plot, the plant community most closely resembled the type initially found at the site U4b (*Festuca ovina-Agrostis capillaris-Galium saxatile* grassland, *Holcus lanatus-Trifolium repens* sub-community), which is typical of relatively fertile but base-poor grasslands (Table 4).

#### Plant species cover and richness

Across the site 37 plant species were recorded, 14 grasses, 14 herbs, 7 legumes and 2 bryophytes; this included 7 species which were each only recorded in one plot. *Holcus lanatus* and *Rumex acetosa* were present in all 14 plots, while *Anthoxanthum odoratum* was recorded in 13 (Table 5).

**Table 5:** Mean species cover of the most common plant species present in each fertilizer treatment plot.

Species	Functional group	Cover (%)														
		Plot number	1	2	3	4	5	6	7	8	9	10	11	12	13	14
		Treatment^a^	FYM +NPK	FYM	FYM +NPK	FYM	FYM +NPK	Ctrl	N	P	K	NP	NK	PK	NPK	NPK
***Agrostis capillaris***	Grass		0	0	0.1	0	0	13	20	11	13	24	34	17	24	24
***Alopecurus pratensis***	Grass		51	44	58	44	51	1	0	21	0	8	0	25	11	5
***Anthoxanthum odoratum***	Grass		0	1	2	13	6	24	64	33	27	38	62	41	27	34
***Bromus hordeaceus***	Grass		30	28	30	23	33	0.1	0	0	0	1	0	8	2	3
***Cynosurus cristatus***	Grass		0	0	0.1	0.1	1	1	0	11	0.1	0	0	1	0	0
***Dactylis glomerata***	Grass		0	0	0	3	7	0.1	0	4	0	1	0	0	2	7
***Festuca rubra***	Grass		0	0	0	1	0.1	72	38	28	65	39	19	12	46	6
***Holcus lanatus***	Grass		33	26	25	18	23	2	9	8	7	14	2	27	18	36
***Lolium perenne***	Grass		0.1	0	1	3	1	0.1	0	3	0.1	1	0	0.1	0	4
***Luzula campestris***	Grass		0	0	0	0	0	2	23	2	6	3	14	0.1	12	0
***Poa trivialis***	Grass		26	32	13	9	9	0	0	0.1	0	1	0	0.1	0	2
***Trisetum flavescens***	Grass		0	0	0	0	0	0	0	0.1	0	0	0	0	0	0
***Anthriscus sylvestris***	Herb		11	13	1	0.1	1	0	0	0	0	0	0	0	0	0
***Bellis perennis***	Herb		0	0	0	0	0	0	0	1	0	0.1	0	1	0	0
***Cerastium fontanum***	Herb		0	0	0.1	2	2	0.1	0	0.1	0	0	0	0	0	0.1
***Conopodium majus***	Herb		0	0	0	0	0	3	0	0	2	0	0	0	0	0.1
***Ficaria verna***	Herb		0	0	0	0.1	0	0	0	1	0	0	0	0	0	0
***Plantago lanceolata***	Herb		0	0	0	0	0	24	0	17	16	10	0	3	1	0
***Ranunculus acris***	Herb		0	1	2	4	1	0.1	0	1	0.1	0.1	0	5	2	0.1
***Ranunculus bulbosus***	Herb		0.1	2	2	3	2	8	0	7	3	5	0	4	1	0.1
***Rhinanthus minor***	Herb		0	0	0	0	0	19	0	19	3	1	0	1	0	0
***Rumex acetosa***	Herb		0.1	2	2	4	4	2	0.1	3	3	8	0.1	8	8	14
***Stellaria media***	Herb		0.1	2	0	0	1	0	0	0	0	0	0	0	0	0
***Taraxacum officinale***	Herb		2	2	0	0	0	0	0	0.1	0	0	0	0.1	0	0
***Lathyrus pratensis***	Legume		0	0	0.1	0.1	0.1	0	0	0.1	0	0	0	2	0	0
***Trifolium pratense***	Legume		0	0	0	0.1	0	12	0	17	10	3	0	7	2	0
***Trifolium repens***	Legume		0	0	0	0	0	3	0	1	0.1	0.1	0	0.1	0.1	0
***Vicia sativa***	Legume		0	0	0	0.1	0	0	0	2	0	0	0	0	0	0
***Vicia sepium***	Legume		0	4	2	1	1	0	0	0	0	0	0	0	0	0

Species with cover <0.5% presented as 0.1%.

^a^ see Table 1 for fertilizer treatment details.

**Fig 6:**
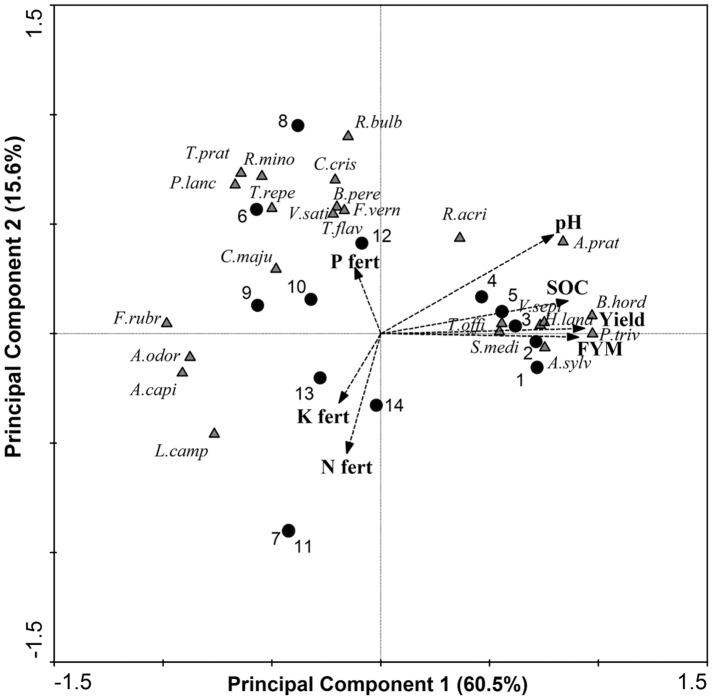
Principal components analysis (PCA) illustrating the variation in the plant species (∆) dataset at Palace Leas. Environmental variables including soil and plant variables (dotted arrow) and treatment plot number (●) are overlain. *A*. *capi = Agrostis capillaris*, *A*. *prat = Alopecurus pratensis*, *A*. *odor = Anthoxanthum odoratum*, *A*. *sylv = Anthriscus sylvestris*, *B*. *pere = Bellis perennis*, *B*. *hord = Bromus hordeaceus*, *C*. *maju = Conopodium majus*, *C*. *cris = Cynosurus cristatus*, *F*. *rubr = Festuca rubra*, *F*. *vern = Ficaria verna*, *H*. *lana = Holcus lanatus*, *L*. *camp = Luzula campestris*, *P*. *triv = Poa trivialis*, *R*. *acri = Ranunculus acris*, *R*.*bulb = Ranunculus bulbosus*, *R*. *mino = Rhinanthus minor*, *S*. *medi = Stellaria media*, *T*. *offi = Taraxacum officinale*, *T*. *prat = Trifolium pratense*, *T*. *repe = Trifolium repens*, *T*. *flav = Trisetum flavescens*, *V*. *sati = Vicia sativa*, *V*. *sepi = Vicia sepium*.

The two dominant axes of the PCA for the percentage cover of the plant species explained 76.1% of the total variance. PC1 accounted for 60.5% of the variation in the data and was strongly positively associated with the application of FYM (r = 0.91). The axis PC2 accounted for 15.6% of the variation in the data and was negatively correlated with the application of N (*r* = -0.55) and K fertilizer (*r* = -0.32) and positively correlated with P fertilizer application (*r* = 0.30; Fig 6). The plant community composition was distinctly different where FYM was applied. The abundance of the grasses *Alopecurus pratensis*, *Bromus hordeaceus*, *Holcus lanatus* and *Poa trivialis* was positively associated with the application of FYM (Fig 6), while the percentage cover of herbs and legumes decreased, with the exception of the tall herb *Anthriscus sylvestris* (Fig 4C and 4D).

Where FYM was not applied and hay yield and soil pH was lower; there was a high abundance of several less vigorous grass species; *Anthoxanthum odoratum*, *Agrostis capillaris* and *Festuca rubra* (Fig 6). The mineral N fertilizer plots (N and NK) had very low plant species richness (5 species per m^2^) (Fig 4E) and herb cover (0.03%), and legumes and bryophytes were absent (Fig 4C and 4D; Table 4). In plots where neither N nor FYM was applied (6; control, 8; P, 9; K, 12; PK), species richness was higher (range 12–18 species per m^-2^), as was herb (range 20.6–57.1%) and legume cover (range 9.3–17.1%) (Fig 4C–4E). The control and P only plots had higher plant species richness (15 and 18 species per m^2^, respectively), herb cover (34.8 and 57.1%, respectively) and legume cover (15.6 and 17.1%, respectively) but the lowest cover of grass species (109.1 and 102.8%, respectively) (Fig 4B–4E). In these plots the cover of herb species *Plantago lanceolata* and *Rhinanthus minor* and legumes including *Trifolium pratense* was high (Table 5). Bryophytes were only present in the control, K and NP treatment plots (Table 5).

## Discussion

### Effects of long-term fertilization on soil pH and nutrient availability

Our results clearly show that very long-term fertilization (120 years) led to marked differences in plant and soil properties between treatment plots in this grassland ecosystem. As hypothesized, many of these differences appear to result from the effects of long-term fertilization on both soil nutrient content and pH, which differed considerably between the plots. In general, in plots where FYM was applied Olsen-P, exchangeable K and soil pH was higher than in plots applied with mineral fertilizer, resulting in increased hay yield. In the long-term Park Grass experiment, levels of Olsen-P and exchangeable K were low in soils applied with only N or the control treatment plots. Olsen-P in these treatment plots at both sites was approximately 3–4 mg P kg^-1^, while on average levels of exchangeable K were higher in these treatment plots at Palace Leas (90 mg K kg^-1^) than Park Grass (60 mg K kg^-1^) [27], potentially due to the inherent high illite content of the soil at Palace Leas [35].

It is thought that soils receiving FYM had a higher buffering capacity to resist acidification due to the presence of Ca^2+^ and Mg^+^ in the manure and/or the oxidation of organic anions during manure decomposition, which go on to consume H^+^ ions [56]. Until now, there has been very little evidence of an effect of FYM on soil pH in long-term field trials. This may be due to the fact that many experiments regularly apply lime to counteract acidification (e.g. Broadbalk and Hoosfield Barley Experiments [57]), meaning the pH buffering effect of FYM goes undetected.

Among the mineral fertilizer treatment plots, soils receiving P but no N maintained a higher pH than in plots where N fertilizer was applied, most likely due to the residual liming properties of the basic slag [38, 58]. In contrast, soils receiving mineral N fertilizer had become highly acidic, no doubt due to acidification associated with the long-term application of ammonium-containing fertilizer [59]. The negative effects of long-term N fertilization on soil pH have also been established at Park Grass where ammonium sulphate addition caused a *c*. 2 unit reduction in soil pH (0–23 cm) in some of the treatment plots [31]. The levels of acidity in the N only and NK treated plots at Palace Leas and their equivalent at Park Grass (plot 1d and 18d) were very similar (Palace Leas 0–10 cm range; pH 4.1–4.2, Park Grass 0–23 cm range; pH 3.9–4.0). After 100 years of ammonium sulphate addition (in 1d and 18d) the soil pH at Park Grass has reached a pH equilibrium [60]. Based on this we anticipate a similar situation in the N only and NK treated plots at Palace Leas, with a further dramatic decline in soil pH unlikely. While greater availability of P and K and higher pH amounted to higher hay yield in FYM applied soils in the long-term, differences in hay yield between plots treated with FYM and those treated with ammonium sulphate were far less pronounced at Palace Leas in the first 5 years following the establishment of the experiment (Fig 5). This was probably due to temporal differences in the drivers of fertilization. In the short-term, plant growth response to fertilization was likely mediated by direct nutrient input, while repeated application of (NH_4_)_2_SO_4_ progressively acidified the soil, resulting in a reduction in nutrient availability [56], and constraining plant growth in the long-term. Consistent with our hypothesis, this demonstrates that there is disparity between short- and long-term ecosystem responses to fertilization and emphasizes the importance of avoiding extrapolating long-term effects from observations made from short-term experiments.

### Effects of long-term fertilization on SOC stocks and particle-size carbon fractions

Long-term FYM addition was associated with an increase in total SOC stocks and greater very fine fraction C stocks, indicative of the mineral-associated C pool, which is typically more stable. We cannot be certain of the mechanism responsible for C accrual in these soils as FYM increases C input and buffers acidification simultaneously, making it difficult to evaluate their individual contribution. However, findings from a recent extensive landscape study suggests that soils with a higher pH within the observed range do not have greater SOC stocks [61]. While an increase in pH has previously been shown to positively influence SOC, including in the Park Grass experiment, results indicate that this is unlikely to occur at pH <6 [61, 62]. It appears more plausible that the positive effects of FYM addition on SOC stocks were due to increased C input via the direct addition of organic matter in FYM (Table 1). Furthermore, because FYM addition also increases plant productivity and hence plant litter residues entering the soil, this could also have increased soil C [35]. C inputs from aboveground litter and root-derived C have been shown to be a key mechanism in building SOC stocks [63, 64].

An increase in SOC stocks following FYM addition as reported here is similar to the trend observed globally. A recent meta-analysis demonstrated that average SOC stock difference was significantly higher where manure was applied (+0.94 kg C ha^-1^) compared to the unfertilized control [65]. At Palace Leas, while SOC stock differences between plots treated with manure only (plot 2 and 4) and the control plot was positive, it was considerably lower (+0.44 kg C ha^-1^) than the global average. Lower soil C accrual may be explained by a number of factors, including differences in soil texture, climatic conditions or land use and management. However, Maillard and Angers [65] established that with the exception of climate, which would be expected to favour soil C accumulation at Palace Leas due to the cool temperate climate retarding SOC decomposition, the effects of manure addition on SOC stocks were independent of these explanatory factors. Alternatively, and congruent with our hypothesis the discrepancy may have arisen as a consequence of differences in experiment length. SOC stocks at Palace Leas are at or close to equilibrium [35] due to over a century of continuous management, which may not be the case in shorter term studies included in the meta-analysis (average 18 years) and which may have included studies recovering from tillage. In short-term experiments, C stocks in both manured and control treatments are likely to fluctuate, hence a transient SOC stock difference may be observed. Once stocks have stabilized, the difference between SOC stocks in the FYM applied and the control treatments may be closer to the figure reported at Palace Leas.

The accumulation of SOC at the soil surface where pH was <4 was strongly associated with increased acidity (Fig 3). Results indicate that much of this C was stored in the coarse and fine carbon fractions, rather than being protected against microbial decomposition as mineral-associated carbon. Given that pH is a key determinant of microbial activity [66], intense acidification is likely to have reduced rates of litter and SOC decomposition [17] and encouraged acid tolerant but slower growing plants with slowly decomposing tissues, both of which would instigate the build-up of organic matter at the soil surface. In the _H_NPK treated plot high hay yields and therefore litter inputs may have operated additively to acidification effects to increase C stocks at 0–5 cm.

Our findings are broadly consistent with the long-term Park Grass experiment, where organic matter decomposition also appeared to be retarded by N-induced acidification, similarly resulting in an increase in organic matter at the soils surface [35]. In contrast, other long-term experiments (37 years [67], >100 years [68]) have found no effect of acidification on SOC stocks. It may be that as these studies sampled deeper soil (0–10 cm and 0–23 cm, respectively) this potentially diluted the trend found here; higher SOC content in the top few centimetres of the profile. It is clear from results presented here that soil properties differed greatly between depths, even within the same treatment plot. Sampling deeper in the soil profile and treating soil as a homogenous sample is becoming more common and therefore the question of depth differences has not been addressed in other long-term experiments. Sampling the soil at finer depth resolution (e.g. in 5 cm layers) may unearth fertilization effects that otherwise could potentially be missed. This is likely to be particularly relevant in grasslands where tillage tends to be less frequent and in acid soils where there is greater vertical stratification [69]. To our knowledge there is no evidence of the magnitude of the acidification effect on SOC observed at Palace Leas and Park Grass, broadly equivalent to the early stages of podzol formation, in short-term experiments. However, it is recognized that it can take decades or centuries for SOC to reach an equilibrium following management change [26], which highlights the importance of using long-term experiments to identify the otherwise hidden long-term responses of SOC.

### Effects of long-term fertilization on plant properties

In line with our hypothesis long-term fertilization at Palace Leas resulted in significant variation in botanical composition between the treatment plots, and this is likely to have been caused by the effects of both direct nutrient input and soil pH changes. Both the higher soil nutrient status and also less acidic soil conditions in the plots receiving FYM resulted in enhanced hay yield and shifted the community towards dominance by tall grass species; *Alopecurus pratensis*, *Bromus hordeaceus* and *Holcus lanatus* which are likely to have outcompeted herb and legume species for light [70].

In contrast, plots that received mineral fertilizers (apart from the P only) have shifted to support communities typical of semi-improved acidic grassland. With the exception of the _H_NPK treated plot these were low yielding and dominated by short, acid tolerant species including *Anthoxanthum odoratum*, *Agrostis capillaris*, *Festuca rubra and Luzula campestris*. Among these communities, the N only and NK treated plots had very low species richness, while legumes and bryophytes were absent. Like the Park Grass and Steinach Grassland experiments, where species richness was lowest in plots receiving (NH_4_)_2_SO_4_, it is probable that the cause of the extreme decline in species richness at Palace Leas was also primarily due to soil acidification [31, 32], which has been shown previously to reduce the availability of base cations, including K^+^ and Mg^2+^ [71], and P availability [72] and increase toxic metal concentrations.

Despite intense acidification in the plot receiving _H_NPK, this plot produced high hay yield and a sward including potentially tall but acid tolerant grass species e.g. *Holcus lanatus*. A high hay yield response in the _H_NPK plot indicates that the positive effect of nutrient input on plant growth overwhelmed the negative impact of soil acidification in this plot. Unlike the other very acidic treatmnt plots, low species richness here was likely to be the combined result of both light exclusion and soil acidification [28]. These responses of species abundances to long-term fertilization are consistent with reports from the Park Grass and Steinach Grassland experiments where in intensely acidic soils very few species (e.g. *Anthoxanthum odoratum* and *Holcus lanatus*) could endure the extreme acidic conditions and toxic concentrations of Al [31].

Unfertilized and P only plots contained plant community types typical of traditionally managed and unfertilized meadows. These were also low yielding plots with a low number of tall grass species but a high abundance of shorter growing herbs such as, *Plantago lanceolata* and legumes including *Trifolium pratense*. These plots also contained the keystone hemiparasite *Rhinanthus minor*, which is known to supress the dominance of tall grasses [73], boost plant species diversity and alter rates of N cycling of the communities it contains [74]. Results from Park Grass support these findings and show that where N was not applied, species richness and the cover of legumes was higher. However, in contrast to Park Grass, species richness was marginally higher in the P only plots at Palace Leas, rather than the control [31, 75]. This response was unlikely to be due to an increase in P availability as high species richness has often been associated with low levels of soil P [76]. It seems more reasonable to suggest that this was due to pH differences as P only plots (pH 4.9) have a higher pH than the control (pH 4.6). Differences in soil pH may also explain why the total species richness in unfertilized control plots at Park Grass (plot 3d; pH 5.2, plot 12d; pH 5.1) were considerably greater (3d; n = 36 species, 12d; n = 42 species) [31] than that observed at Palace Leas (n = 22 species).

The distinct patterns in the plant community dynamics displayed here and at other long-term fertilized sites are unlikely to be accurately predicted in short-term studies [27]. For example, in lowland semi-improved and unimproved grasslands in Wales, which received 24 t FYM ha^-1^ y^-1^ for 7 years, plant species density ((mean response in the fertilizer treatment/ mean response in the unfertilized control)[3]) was reduced by 10% and 21%, respectively [77]. Long-term FYM application at approximately the same rate at Palace Leas (plot 2; 20 t FYM ha^-1^ y^-1^) caused plant species density to be reduced by 43%, a considerably greater loss of plant species compared to this short-term study. Similarly, in a meta-analysis using data compiled from relatively short-term studies (4–15 years), plant species density was reduced on average by 28% in response to N fertilization [3], whereas at Palace Leas in the plot applied with only N fertilizer ((NH_4_)_2_SO_4_) there was a 66% reduction in species density. These findings indicate that while the direction of short- and long-term fertilization effects may concur, the magnitude of the effects are markedly different, particularly where long-term ammonium sulphate addition caused soil acidification. While the magnitude of the effect of fertilization may increase with experimental length meaning the pattern observed at Palace Leas may have been more accurate of the long-term response, we acknowledge that the variation in the magnitude of the effect between studies of different duration may also be a result of other factors, such as; differences in experimental design, community-specific mechanisms [3] and the species pool able to colonize the treatment plot [78].

Despite the fact that species loss and compositional change has been reported in nutrient addition experiments of varying duration [12, 31], the occurrence of a dramatic change in the vegetation community and species loss as observed here and at Park Grass [79] was potentially a consequence of very long-term fertilization. A dramatic change in plant community composition, also known as a regime shift, occurs once a critical environmental threshold has been surpassed causing a shift in the community to an alternative stable state. However, the duration of short-term studies may not be sufficient to detect such phenomena, hence without the use of long-term experiments these events are likely to be missed [79, 80].

### Implications of the findings

While global agricultural productivity is heavily dependent on the use of fertilizers [81] our results demonstrate that their long-term addition can strongly affect several other ecosystem services. In terms of agricultural production, our results suggest that the addition of ammonium sulphate over long periods of time can cause severe acidification and significantly constrain crop productivity compared to where FYM is applied. An increase in soil acidification is likely to reduce rates of nitrification, solubility of P and base cation availability [8, 56, 82], indicating that crop productivity is unlikely to be maintained by mineral fertilizer addition in the long-term and will not be economically viable for the farmer. Our results also suggest that the long-term addition of N-containing fertilizers (both FYM and mineral fertilizer) will strongly reduce plant diversity, such effects may also reduce the diversity of a range of vertebrate and invertebrate taxa not measured here [83]. Attempts to remediate these effects could involve ceasing fertilizer applications and in areas of intense acidity the addition of lime to increase soil pH. Indeed, a recent study at Park Grass reported that by liming and the cessation of nutrient addition, over time grasslands have the capacity to successfully reverse the negative effects of long-term fertilization on plant species diversity [84]. However, the extent of remediation may be compromised where long-term ammonium sulphate addition causes intense acidity as grassland recovery is considerably slower [84]. In light of this we suggest that farmers should maximize nutrient use from FYM to reduce detrimental implications to ecosystem services, lower investment in mineral fertilizers and so that soil C gains may be realized. While the use of ammonium sulphate is now less common than in the 20^th^ Century, we suggest even in situations similar to Palace Leas, where N application rate was relatively low, that soil pH be monitored closely from the onset of fertilization and periodic liming is undertaken to correct a pH decline and avert the impacts of acidification, rather than after the fact, as grassland recovery is likely to be slow. Our results also underline the importance of sampling the soil profile with appropriate resolution with depth, in particular in acid grassland soils which are susceptible to vertical stratification. By using this approach, we were able to establish that there were similar patterns in SOC stocks at low pH as those reported at Park Grass, which in other long-term studies may have been missed. As it often requires decades for ecosystem properties to stabilize in response to fertilization, short-term experiments may only detect transient or intermediate effects of fertilization upon ecosystem properties. This, coupled with strong consistencies in patterns of soil and plant response between long-term fertilizer studies firmly emphasizes the importance of utilising long-term experiments to better understand long-term ecosystem responses. However, approaches such as those used here remain somewhat phenomenological, making our understanding of the relative importance of the pathways and mechanisms that drive ecosystem responses to fertilization poor. Thus, more attention is required to investigate the relative importance of direct nutrient, pH and plant community composition mediated pathways on grassland ecosystem properties in the future.

While findings from long-term experiments clearly still have practical and biological implications for grassland management, the majority of such experiments were established at a time when management methods and the intentions of their creators were very different to the approaches used and the questions posed by the scientific community today. There is a now a need for new long-term nutrient addition experiments to be established, which take into consideration current and expected trends in fertilizer management and existing medium-term experiments to be maintained. Currently, the majority of the world’s longest continuously managed trials are situated in the USA and across Europe. However, projections suggest that the greatest increases in fertilization will occur in China, India and other developing countries [2] where the impacts of fertilization are relatively unknown. Therefore, a strategy must be developed to establish an economically secure network of long-term experiments [79] across a range of environmental and climatic gradients, and particular in areas where plant diversity is high [85], the provision of other ecosystem service are important and fertilizer use is predicted to be most intense.

## Supporting information

S1 Table Soil pH, organic carbon stocks (SOC), total nitrogen content, soil carbon to nitrogen ratio (C/N), coarse fraction carbon stocks, fine fraction carbon stocks, very fine fraction carbon stocks and exchangeable Al for the O horizon in treatment plots 7 and 11. Values are means (±1 SE), n/a indicates not applicable.
(DOCX)

S1 Dataset Data on soil and plant properties and hay yield used for the analysis. (XLSX)
