# Supplementary material for: Impacts of 120 years of fertilizer addition on a temperate grassland ecosystem
Source: PLoS One. 2017 Mar 28;12(3):e0174632. doi: 10.1371/journal.pone.0174632 (PMC5369769; doi:10.1371/journal.pone.0174632)
Supplement: S1 Table — Values are means (±1 SE), n/a indicates not applicable. (DOCX) [file pone.0174632.s001.docx]

| **Plot number** | **Treatment** | **Depth (cm)** | **pH** | **SOC**  **(kg C m^-2^)** | **Total nitrogen**  **content**  **(kg N m^-2^)** | **Soil C/N** | **Coarse fraction carbon stocks (kg C m^-2^)** | **Fine fraction carbon stocks (kg C m^-2^)** | **Very fine fraction carbon stocks**  **(kg C m^-2^)** | **Exchangeable Al**  **(mmol kg^-1^)** |
| --- | --- | --- | --- | --- | --- | --- | --- | --- | --- | --- |
| 7 | N | O horizon | 3.26 (0.03) | 3.69 (0.15) | 0.24 (0.03) | 14.45 (0.67) | 1.17 (0.16) | 1.59 (0.17) | 0.64 (0.15) | 37.64 |
| 11 | NK | O horizon | 3.17 (0.06) | 3.59 (0.13) | 0.31 (0.05) | 14.42 (0.68) | 0.50 (0.11) | 2.50 (0.23) | 1.12 (0.14) | 37.78 |
